# *N*′-(3,4-Dihydroxy­benzyl­idene)acetohydrazide

**DOI:** 10.1107/S1600536809030712

**Published:** 2009-08-08

**Authors:** Wei-Wei Li, Lu-Ping Lv, Wen-Bo Yu, Yong-Zhao Zhang, Xian-Chao Hu

**Affiliations:** a Department of Chemical Engineering, Hangzhou Vocational and Technical College, Hangzhou 310018, People’s Republic of China; b Research Center of Analysis and Measurement, Zhejiang University of Technology, Hangzhou 310014, People’s Republic of China

## Abstract

In the title compound, C_9_H_10_N_2_O_3_, the Schiff base mol­ecule is approximately planar, the dihedral angle between the benzene ring and the acetohydrazide group (r.m.s. deviation = 0.034 Å) being 8.81 (7)°. An intra­molecular O—H⋯O hydrogen bond is observed.  In the crystal, mol­ecules are linked into a three-dimensional network by O—H⋯O, N—H⋯O and C—H⋯O hydrogen bonds.

## Related literature

For general background to Schiff bases, see: Cimerman *et al.* (1997 ▶); Offe *et al.* (1952 ▶); Richardson *et al.* (1988 ▶). For related structures, see: Li *et al.* (2008 ▶); Tamboura *et al.* (2009 ▶).
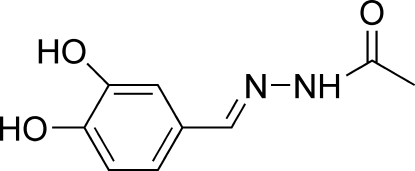


## Experimental

### 

#### Crystal data

C_9_H_10_N_2_O_3_
*M* *_r_* = 194.19
Monoclinic, 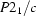

*a* = 10.598 (2) Å
*b* = 8.5017 (16) Å
*c* = 10.621 (2) Å
β = 107.232 (7)°
*V* = 913.9 (3) Å^3^
*Z* = 4
Mo *K*α radiation
μ = 0.11 mm^−1^
*T* = 223 K
0.25 × 0.24 × 0.20 mm

#### Data collection

Bruker SMART CCD area-detector diffractometer
Absorption correction: multi-scan (*SADABS*; Bruker, 2002 ▶) *T*_min_ = 0.972, *T*_max_ = 0.980
5752 measured reflections
2066 independent reflections
1665 reflections with *I* > 2σ(*I*)
*R*_int_ = 0.023

#### Refinement

*R*[*F* ^2^ > 2σ(*F* ^2^)] = 0.037
*wR*(*F* ^2^) = 0.111
*S* = 0.94
2066 reflections
129 parameters
H-atom parameters constrained
Δρ_max_ = 0.21 e Å^−3^
Δρ_min_ = −0.17 e Å^−3^

### 

Data collection: *SMART* (Bruker, 2002 ▶); cell refinement: *SAINT* (Bruker, 2002 ▶); data reduction: *SAINT*; program(s) used to solve structure: *SHELXS97* (Sheldrick, 2008 ▶); program(s) used to refine structure: *SHELXL97* (Sheldrick, 2008 ▶); molecular graphics: *SHELXTL* (Sheldrick, 2008 ▶); software used to prepare material for publication: *SHELXTL*.

## Supplementary Material

Crystal structure: contains datablocks I, global. DOI: 10.1107/S1600536809030712/ci2878sup1.cif

Structure factors: contains datablocks I. DOI: 10.1107/S1600536809030712/ci2878Isup2.hkl

Additional supplementary materials:  crystallographic information; 3D view; checkCIF report

## Figures and Tables

**Table 1:** Hydrogen-bond geometry (Å, °)

*D*—H⋯*A*	*D*—H	H⋯*A*	*D*⋯*A*	*D*—H⋯*A*
N2—H2⋯O1^i^	0.86	2.17	2.9692 (15)	154
O2—H2*A*⋯O1^ii^	0.82	1.96	2.7206 (13)	154
O3—H3⋯O2	0.82	2.26	2.7109 (14)	115
O3—H3⋯O2^iii^	0.82	2.14	2.7784 (14)	134
C9—H9*C*⋯O3^iv^	0.96	2.51	3.445 (2)	166

Symmetry codes: (i) 
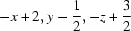
; (ii) 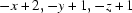; (iii) 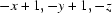; (iv) 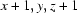.

**Fig. 1.**
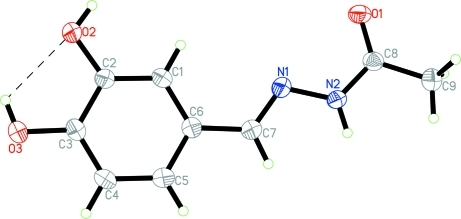
The molecular structure of the title compound. Displacement ellipsoids are drawn at the 40% probability level. The dashed line indicates a hydrogen bond.

**Fig. 2.**
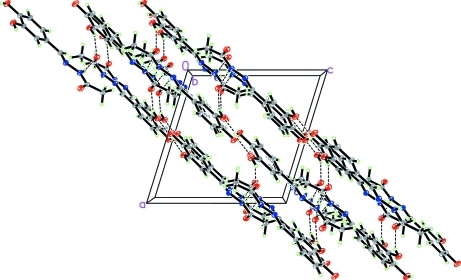
Part of the crystal packing of the title compound. Hydrogen bonds are shown as dashed lines.

## supplementary crystallographic information

### Comment

Schiff bases have attracted much attention due to the possibility of their analytical applications (Cimerman *et al.*,  1997). They are also important ligands, which have been reported to have mild bacteriostatic activity and as potential oral iron-chelating drugs for genetic disorders such as thalassemia (Offe *et al.*, 1952; Richardson *et al.*,  1988). Metal complexes based on Schiff bases have received considerable attention because they can be utilized as model compounds of active centres in various complexes (Tamboura *et al.*,  2009). We report here the crystal structure of the title compound (Fig. 1).

In the Schiff base molecule, the acetohydrazide group (O1/N1/N2/C8/C9) is planar (r.m.s. deviation 0.034 Å) and it forms a dihedral angle of 8.81 (7)° with the benzene (C1-C6) ring. The molecule adopts a *trans* configuration with respect to the C═N bond. Bond lengths and angles are comparable to those observed for *N*'-[1-(4-methoxyphenyl)ethylidene]acetohydrazide (Li *et al.*, 2008). An intramolecular O3—H3···O2 hydrogen bond is observed.

In the crystal, molecules are linked into a three-dimensional network (Fig.2) by O—H···O, N—H···O and C—H···O hydrogen bonds (Table 1).

### Experimental

3,4-Dihydroxybenzaldehyde (1.38 g, 0.01 mol) and acetohydrazide (0.74 g, 0.01 mol) were dissolved in stirred methanol (20 ml) and left for 2.5 h at room temperature. The resulting solid was filtered off and recrystallized from ethanol to give the title compound in 85% yield. Single crystals suitable for X-ray analysis were obtained by slow evaporation of an ethanol solution at room temperature (m.p. 475–477 K).

### Refinement

H atoms were positioned geometrically (N-H = 0.86 Å, O-H = 0.82Å and C-H = 0.93 or 0.96Å) and refined using a riding model, with *U*_iso_(H) =1.2*U*_eq_(C,N) and 1.5*U*_eq_(C_methyl_).

### Crystal data

**Table:**

C_9_H_10_N_2_O_3_	*F*(000) = 408
*M**_r_* = 194.19	*D*_x_ = 1.411 Mg m^−^^3^
Monoclinic, *P*2_1_/*c*	Mo *K*α radiation, λ = 0.71073 Å
Hall symbol: -P 2ybc	Cell parameters from 2066 reflections
*a* = 10.598 (2) Å	θ = 2.0–27.4°
*b* = 8.5017 (16) Å	µ = 0.11 mm^−^^1^
*c* = 10.621 (2) Å	*T* = 223 K
β = 107.232 (7)°	Block, colourless
*V* = 913.9 (3) Å^3^	0.25 × 0.24 × 0.20 mm
*Z* = 4	

### Data collection

**Table:**

Bruker SMART CCD area-detector diffractometer	2066 independent reflections
Radiation source: fine-focus sealed tube	1665 reflections with *I* > 2σ(*I*)
graphite	*R*_int_ = 0.023
φ and ω scans	θ_max_ = 27.4°, θ_min_ = 2.0°
Absorption correction: multi-scan (*SADABS*; Bruker, 2002)	*h* = −13→13
*T*_min_ = 0.972, *T*_max_ = 0.980	*k* = −10→10
5752 measured reflections	*l* = −13→12

### Refinement

**Table:**

Refinement on *F*^2^	Secondary atom site location: difference Fourier map
Least-squares matrix: full	Hydrogen site location: inferred from neighbouring sites
*R*[*F*^2^ > 2σ(*F*^2^)] = 0.037	H-atom parameters constrained
*wR*(*F*^2^) = 0.111	*w* = 1/[σ^2^(*F*_o_^2^) + (0.0674*P*)^2^ + 0.2108*P*] where *P* = (*F*_o_^2^ + 2*F*_c_^2^)/3
*S* = 0.94	(Δ/σ)_max_ = 0.009
2066 reflections	Δρ_max_ = 0.21 e Å^−^^3^
129 parameters	Δρ_min_ = −0.17 e Å^−^^3^
0 restraints	Extinction correction: *SHELXL97* (Sheldrick, 2008), Fc^*^=kFc[1+0.001xFc^2^λ^3^/sin(2θ)]^-1/4^
Primary atom site location: structure-invariant direct methods	Extinction coefficient: 0.019 (4)

### Special details

**Table:**

Geometry. All esds (except the esd in the dihedral angle between two l.s. planes) are estimated using the full covariance matrix. The cell esds are taken into account individually in the estimation of esds in distances, angles and torsion angles; correlations between esds in cell parameters are only used when they are defined by crystal symmetry. An approximate (isotropic) treatment of cell esds is used for estimating esds involving l.s. planes.
Refinement. Refinement of F^2^ against ALL reflections. The weighted R-factor wR and goodness of fit S are based on F^2^, conventional R-factors R are based on F, with F set to zero for negative F^2^. The threshold expression of F^2^ > 2sigma(F^2^) is used only for calculating R-factors(gt) etc. and is not relevant to the choice of reflections for refinement. R-factors based on F^2^ are statistically about twice as large as those based on F, and R- factors based on ALL data will be even larger.

### Fractional atomic coordinates and isotropic or equivalent isotropic displacement parameters (Å^2^)

**Table:**

	*x*	*y*	*z*	*U*_iso_*/*U*_eq_	
N2	1.00361 (10)	0.13127 (13)	0.68707 (10)	0.0357 (3)	
H2	0.9880	0.0352	0.7029	0.043*	
O3	0.47733 (10)	0.25734 (12)	0.01091 (10)	0.0522 (3)	
H3	0.4848	0.3492	−0.0093	0.078*	
O2	0.64301 (9)	0.48688 (11)	0.14025 (10)	0.0424 (3)	
H2A	0.6973	0.5473	0.1870	0.064*	
O1	1.13124 (9)	0.34588 (11)	0.75557 (10)	0.0445 (3)	
N1	0.93041 (10)	0.20010 (13)	0.56882 (11)	0.0362 (3)	
C6	0.75370 (12)	0.15246 (15)	0.36828 (13)	0.0345 (3)	
C3	0.56800 (13)	0.22557 (16)	0.12889 (13)	0.0374 (3)	
C1	0.74814 (12)	0.30536 (15)	0.31694 (13)	0.0338 (3)	
H1	0.8063	0.3821	0.3628	0.041*	
C2	0.65615 (12)	0.34155 (15)	0.19818 (12)	0.0333 (3)	
C5	0.66449 (13)	0.03947 (16)	0.29930 (14)	0.0397 (3)	
H5	0.6673	−0.0617	0.3334	0.048*	
C8	1.09751 (12)	0.21065 (15)	0.77666 (13)	0.0336 (3)	
C9	1.15560 (15)	0.12587 (18)	0.90450 (13)	0.0440 (3)	
H9A	1.1378	0.0153	0.8919	0.066*	
H9B	1.1169	0.1653	0.9693	0.066*	
H9C	1.2494	0.1427	0.9342	0.066*	
C7	0.84748 (13)	0.10590 (15)	0.49377 (13)	0.0359 (3)	
H7	0.8468	0.0018	0.5205	0.043*	
C4	0.57178 (14)	0.07602 (16)	0.18054 (14)	0.0412 (3)	
H4	0.5122	0.0000	0.1357	0.049*	

### Atomic displacement parameters (Å^2^)

**Table:**

	*U*^11^	*U*^22^	*U*^33^	*U*^12^	*U*^13^	*U*^23^
N2	0.0383 (6)	0.0271 (6)	0.0362 (6)	−0.0028 (4)	0.0024 (5)	0.0041 (4)
O3	0.0513 (6)	0.0409 (6)	0.0465 (6)	−0.0082 (4)	−0.0132 (5)	0.0037 (4)
O2	0.0409 (5)	0.0333 (5)	0.0426 (6)	−0.0053 (4)	−0.0036 (4)	0.0054 (4)
O1	0.0454 (5)	0.0301 (5)	0.0501 (6)	−0.0069 (4)	0.0019 (4)	0.0001 (4)
N1	0.0360 (6)	0.0332 (6)	0.0354 (6)	0.0007 (4)	0.0045 (5)	0.0048 (4)
C6	0.0347 (6)	0.0349 (7)	0.0326 (7)	−0.0013 (5)	0.0081 (5)	0.0000 (5)
C3	0.0341 (7)	0.0385 (7)	0.0347 (7)	−0.0021 (5)	0.0024 (5)	−0.0019 (5)
C1	0.0322 (6)	0.0320 (7)	0.0345 (7)	−0.0046 (5)	0.0060 (5)	−0.0027 (5)
C2	0.0326 (6)	0.0304 (6)	0.0350 (7)	−0.0018 (5)	0.0070 (5)	−0.0001 (5)
C5	0.0436 (7)	0.0323 (7)	0.0402 (7)	−0.0048 (5)	0.0079 (6)	0.0020 (5)
C8	0.0332 (6)	0.0288 (6)	0.0376 (7)	0.0014 (5)	0.0086 (5)	−0.0027 (5)
C9	0.0470 (8)	0.0416 (8)	0.0376 (8)	0.0004 (6)	0.0038 (6)	0.0022 (6)
C7	0.0384 (7)	0.0315 (7)	0.0363 (7)	−0.0022 (5)	0.0087 (5)	0.0024 (5)
C4	0.0406 (7)	0.0355 (7)	0.0416 (8)	−0.0097 (5)	0.0031 (6)	−0.0047 (6)

### Geometric parameters (Å, °)

**Table:**

N2—C8	1.3380 (16)	C3—C4	1.381 (2)
N2—N1	1.3944 (14)	C3—C2	1.4069 (18)
N2—H2	0.86	C1—C2	1.3811 (18)
O3—C3	1.3612 (16)	C1—H1	0.93
O3—H3	0.82	C5—C4	1.3851 (19)
O2—C2	1.3689 (16)	C5—H5	0.93
O2—H2A	0.82	C8—C9	1.4989 (19)
O1—C8	1.2438 (16)	C9—H9A	0.96
N1—C7	1.2783 (17)	C9—H9B	0.96
C6—C5	1.3939 (18)	C9—H9C	0.96
C6—C1	1.4041 (18)	C7—H7	0.93
C6—C7	1.4615 (18)	C4—H4	0.93
			
C8—N2—N1	121.80 (11)	C4—C5—C6	120.78 (13)
C8—N2—H2	119.1	C4—C5—H5	119.6
N1—N2—H2	119.1	C6—C5—H5	119.6
C3—O3—H3	109.5	O1—C8—N2	122.07 (12)
C2—O2—H2A	109.5	O1—C8—C9	123.01 (12)
C7—N1—N2	113.26 (11)	N2—C8—C9	114.90 (12)
C5—C6—C1	119.34 (12)	C8—C9—H9A	109.5
C5—C6—C7	117.70 (12)	C8—C9—H9B	109.5
C1—C6—C7	122.94 (12)	H9A—C9—H9B	109.5
O3—C3—C4	118.55 (12)	C8—C9—H9C	109.5
O3—C3—C2	121.38 (12)	H9A—C9—H9C	109.5
C4—C3—C2	120.06 (12)	H9B—C9—H9C	109.5
C2—C1—C6	119.86 (12)	N1—C7—C6	123.72 (12)
C2—C1—H1	120.1	N1—C7—H7	118.1
C6—C1—H1	120.1	C6—C7—H7	118.1
O2—C2—C1	124.19 (11)	C3—C4—C5	119.85 (12)
O2—C2—C3	115.73 (11)	C3—C4—H4	120.1
C1—C2—C3	120.09 (12)	C5—C4—H4	120.1
			
C8—N2—N1—C7	178.32 (12)	C7—C6—C5—C4	179.18 (13)
C5—C6—C1—C2	−0.99 (19)	N1—N2—C8—O1	−5.29 (19)
C7—C6—C1—C2	−179.44 (12)	N1—N2—C8—C9	173.18 (11)
C6—C1—C2—O2	179.96 (12)	N2—N1—C7—C6	176.10 (11)
C6—C1—C2—C3	0.13 (19)	C5—C6—C7—N1	−176.99 (13)
O3—C3—C2—O2	0.93 (19)	C1—C6—C7—N1	1.5 (2)
C4—C3—C2—O2	−178.75 (12)	O3—C3—C4—C5	178.87 (13)
O3—C3—C2—C1	−179.22 (12)	C2—C3—C4—C5	−1.4 (2)
C4—C3—C2—C1	1.1 (2)	C6—C5—C4—C3	0.6 (2)
C1—C6—C5—C4	0.7 (2)		

### Hydrogen-bond geometry (Å, °)

**Table:**

*D*—H···*A*	*D*—H	H···*A*	*D*···*A*	*D*—H···*A*
N2—H2···O1^i^	0.86	2.17	2.9692 (15)	154
O2—H2A···O1^ii^	0.82	1.96	2.7206 (13)	154
O3—H3···O2	0.82	2.26	2.7109 (14)	115
O3—H3···O2^iii^	0.82	2.14	2.7784 (14)	134
C9—H9C···O3^iv^	0.96	2.51	3.445 (2)	166

Symmetry codes: (i) −*x*+2, *y*−1/2, −*z*+3/2; (ii) −*x*+2, −*y*+1, −*z*+1; (iii) −*x*+1, −*y*+1, −*z*; (iv) *x*+1, *y*, *z*+1.
